# Early SARS-CoV-2 dynamics and immune responses in unvaccinated participants of an intensely sampled longitudinal surveillance study

**DOI:** 10.1038/s43856-022-00195-4

**Published:** 2022-10-11

**Authors:** Manjula Gunawardana, Simon Webster, Sofia Rivera, John M. Cortez, Jessica Breslin, Cristian Pinales, Christopher Buser, F. Javier Ibarrondo, Otto O. Yang, Michael Bobardt, Philippe A. Gallay, Amy P. Adler, Christina M. Ramirez, Peter A. Anton, Marc M. Baum

**Affiliations:** 1 Department of Chemistry, Oak Crest Institute of Science, 128–132W. Chestnut Ave., Monrovia, CA USA; 2 University of California, Los Angeles (UCLA), Division of Infectious Diseases, Department of Medicine, David Geffen School of Medicine at UCLA, Los Angeles, CA USA; 3 University of California, Los Angeles (UCLA), Department of Microbiology, Immunology, and Molecular Genetics, David Geffen School of Medicine at UCLA, Los Angeles, CA USA; 4 Department of Immunology and Microbiology, The Scripps Research Institute, La Jolla, CA USA; 5 Jumpstart Research Consulting, LLC, Santa Fe, NM USA; 6 University of California, Los Angeles (UCLA), Department of Biostatistics, Fielding School of Public Health, UCLA, Los Angeles, CA USA

**Keywords:** Medical research, Viral infection, Viral infection

## Abstract

**Background:**

A comprehensive understanding of the SARS-CoV-2 infection dynamics and the ensuing host immune responses is needed to explain the pathogenesis as it relates to viral transmission. Knowledge gaps exist surrounding SARS-CoV-2 in vivo kinetics, particularly in the earliest stages after exposure.

**Methods:**

An ongoing, workplace clinical surveillance study was used to intensely sample a small cohort longitudinally. Nine study participants who developed COVID-19 between November, 2020 and March, 2021 were monitored at high temporal resolution for three months in terms of viral loads as well as associated inflammatory biomarker and antibody responses. CD8 + T cells targeting SARS-CoV-2 in blood samples from study participants were evaluated.

**Results:**

Here we show that the resulting datasets, supported by Bayesian modeling, allowed the underlying kinetic processes to be described, yielding a number of unexpected findings. Early viral replication is rapid (median doubling time, 3.1 h), providing a narrow window between exposure and viral shedding, while the clearance phase is slow and heterogeneous. Host immune responses different widely across participants.

**Conclusions:**

Results from our small study give a rare insight into the life-cycle of COVID-19 infection and hold a number of important biological, clinical, and public health implications.

## Plain language summary

Managing the response to the COVID-19 pandemic requires information about how quickly the virus reproduces and the effect on the immune system of the person who is infected. We measured the speed at which SARS-CoV-2 reproduces in unvaccinated individuals at various timepoints between when they first became infected, and there was no longer any detectable virus present in their bodies. We also measured changes in their immune response. Our findings can be used to develop guidelines for the clinical management of COVID-19 patients and optimize testing procedures to determine whether people are infected with SARS-CoV-2.

## Abstract (web summary)

Gunawardana et al. monitor the viral load, inflammatory biomarkers and antibody response long-term in people who developed COVID-19. Early viral replication is rapid, providing a narrow window between exposure and viral shedding.

## Introduction

A comprehensive understanding of early infection viral dynamics and associated host immune responses is key to describing the underlying disease pathogenesis and is needed to inform effective public health measures and clinical management policies. Characterizing the viral load kinetics in a number of diverse patient populations also can be instrumental in developing new antiviral drugs and therapies. Advances in the management of acute or chronic viral diseases—such as influenza^1^, human immunodeficiency virus (HIV)^2,3^, and hepatitis C virus (HCV)^4^—were aided by foundational studies on clinical viral dynamics. There remain a number of knowledge gaps surrounding severe acute respiratory syndrome coronavirus 2 (SARS-CoV-2) kinetics in coronavirus disease 2019 (COVID-19) patients, and how the viral dynamics interplay with disease progression. Infections with SARS-CoV-2 can be described by two main stages—the viral proliferation and clearance phases—that typically end with a long tail of low-level, persistent viral RNA shedding.

A number of longitudinal clinical studies have examined the SARS-CoV-2 clearance phase^5–9^ after the establishment of infection, but little is known about the rapid, exponential proliferation (i.e., viral growth) phase after exposure. Characterization of the early phase of the viral life-cycle is challenging due to its occurrence before symptoms, if any, and its short duration. Prospective, observational clinical studies to investigate this phenomenon would require large participant cohorts committed to frequent serial sampling, which is logistically difficult.

Controlled human challenge studies have been successful at elucidating the viral kinetics of milder diseases than COVID-19, where effective treatment strategies were available, such as human influenza^1^. In this model, volunteers are deliberately exposed to an infectious challenge agent to study the subsequent infection and the potential benefits of experimental interventions (e.g., antiviral agents, vaccine candidates). Human challenge studies using SARS-CoV-2 could overcome some of the practical limitations of observational clinical studies as participants would be closely monitored in a controlled setting. However, given our limited understanding of COVID-19 and the potential for significant morbidity associated with acute disease presentation as well as persistent, long-lasting symptoms (i.e., so-called “long-COVID”), human challenge studies involving SARS-CoV-2 are controversial and face an ethical dilemma that has been the subject of considerable debate^10–14^. Controlled infection models also suffer from a number of scientific limitations borne out of their inherent artificial nature, such as the choice of the viral strain, the size of the viral inoculum, the mode of inoculation, and the age of the participants, as only young and healthy subjects typically can be enrolled. Two SARS-CoV-2 human challenge studies are ongoing in the United Kingdom^15^. One study reported that 18 out of 34 volunteers (aged 18–29 years) became infected following intranasal inoculation with wild-type virus (SARSCoV-2/human/GBR/484861/2020), and no serious safety signals were detected^16^.

Deepening our nascent understanding of the SARS-CoV-2 dynamics can hold important implications for managing the pandemic. For example, an effective strategy for curbing the spread of SARS-CoV-2 relies on the rapid, early identification of infected individuals followed by isolation. Test-based screening is playing a critical role in these efforts, as symptom presentation is not a reliable indicator of infectiousness^17^. Since March 23, 2020, we have been conducting a continuous, ongoing workplace clinical study involving the longitudinal and intensive characterization of COVID-19 prevalence and incidence at the Oak Crest Institute of Science (Oak Crest), a nonprofit scientific research organization in Southern California. The intensely sampled observational surveillance study has enabled unvaccinated participants who developed COVID-19 to be identified in the early stages of the viral proliferation phase and allowed them to be followed at high temporal resolution. The kinetics of SARS-CoV-2 production and clearance, along with the concomitant host immune responses, reported here hold a number of important biological, clinical, and public health implications, as discussed in detail below.

## Methods

### Ethics statement

All human research under OCIS-05, “Longitudinal Characterization of COVID-19 Prevalence and Incidence in a Small Working Institution with Both Public Health and Diagnostic Aims”, was approved by Aspire IRB (Aspire Study # 1281548) and conducted according to the Declaration of Helsinki. All study participants provided written informed consent or assent. There were some minors participating in the study who were capable of understanding and signing an IRB-approved assent form in addition to the IRB informed consent form completed by their parent or legal guardian.

### Clinical study design

The workplace SARS-CoV-2 surveillance clinical study was initiated by the Oak Crest Institute of Science (Oak Crest, https://www.oak-crest.org/), a small nonprofit academic science research organization located in Monrovia, CA, on 23 March, 2020, has been running without interruptions and is ongoing at the time of writing. The study design has been described in detail elsewhere^18^. Briefly, all Oak Crest employees, students, and volunteers were asked to participate in the prospective, longitudinal, observational study designed to last 12 weeks, or longer. Those choosing not to participate had no negative employment or finance-related consequences but were asked to work from home exclusively. Household members from the above-described study population also were invited to participate in the study. Swab samples (nasal and oral) were collected between 8:30 and 9:00 AM three times per week—with the exception of periods of low, local SARS-CoV-2 positivity rates where the testing frequency was reduced to twice weekly—from participants needing access to the Oak Crest facilities while they were isolated in their motor vehicles.

### Saliva and blood sample collection and processing

Optional saliva samples were self-collected in Falcon tubes (50 ml) at the participant’s home or in their sealed vehicle, and stool swabs were collected at the participant’s home. Specific written instructions were provided to participants opting to provide these specimens. Blood (5–8 ml, ×2) was collected for cytokine and antibody testing by a licensed phlebotomist using Vacutainer (Becton, Dickinson and Company, Franklin Lakes, NJ) tubes for serum (spray-coated silica) and plasma (spray-coated K_2_EDTA) in the Oak Crest parking lot, while the participant remained comfortably seated in their vehicle.

For the analysis of cellular responses to SARS-CoV-2, blood was collected in vacutainer vials (362753, Becton, Dickinson and Company) by standard venipuncture and centrifuged in a horizontal rotor (i.e., swing-out bucket) for 15 min at 2000 × *g*, room temperature. The peripheral blood mononuclear cell (PBMC) layer aggregated in a whitish layer just under the plasma layer, which was removed to separate vials and stored at −80 °C for future analysis. The PBMC layer was removed into a sterile 15 ml conical centrifuge tube, taking care not to disrupt the separation. Cell media (RPMI 1640, 11835030, Thermo Fisher Scientific, Waltham, MA) was added up to the volume of 10 mL while resuspending the cells. An aliquot (10 µL) was removed for cell counting, and the remaining sample was centrifuged again at 2000 × *g* for 15 min. The resulting supernatant was aspirated, and the PBMC pellet was resuspended in freshly prepared, ice-cold freezing media consisting of dimethyl sulfoxide in fetal bovine serum (10% v/v, 26140079, Thermo Fisher Scientific) to a final concentration of 3.0 × 10^6^ cells mL^−1^. The cellular suspensions were dispensed as 1.0 mL aliquots into pre-chilled, labeled vials. The PBMC samples were stored at −80 °C for 24 h and then transferred to liquid nitrogen storage until use.

### Analysis of clinical nasal swab specimens by transmission electron microscopy (TEM)

Nasal swab samples collected from participants who tested positive for SARS-CoV-2 RNA by RT-qPCR and a SARS-CoV-2 RT-qPCR negative control were stored directly either in glutaraldehyde in PBS (5% v/v) or formaldehyde in PBS (8% w/v). These fixative concentrations were chosen two-fold above our standard mixture to maintain concentrations above accepted SARS-CoV-2 inactivation thresholds under all circumstances. The samples were allowed to react at room temperature for 12 h to further ensure complete virus inactivation and then stored at 4 °C until sample preparation. Swab segments were cut with a razor blade, and formaldehyde-fixed samples were fixed further in glutaraldehyde in PBS (1% v/v). The segments were post-fixed in aqueous osmium tetroxide (2% w/v), block-stained in aqueous uranyl acetate (1% w/v), dehydrated in an ethanol series, and embedded in Spurr’s resin. The resulting blocks were sectioned 50–70 nm thin and collected on formvar filmed 2 × 1 mm slot grids, stained with aqueous uranyl acetate (2% w/v) and Reynolds lead citrate, and imaged at 80 kV in a Model EM10 (Carl Zeiss AG, Oberkochen, Germany) TEM equipped with an Gatan Erlangshen ES1000W (Pleasanton, CA) CCD camera. Images were enhanced for brightness/contrast as needed using ImageJ.

### Measurement of SARS-CoV-2 viral loads in clinical samples by RT-qPCR

The samples (nasal, stool, saliva) were analyzed for SARS-CoV-2 RNA copy numbers by reverse transcription (RT) and quantitative PCR (qPCR) using primer sequences targeting the SARS-CoV-2 nucleocapsid protein (*N*) gene transcript fragments (N1 and N2) and one human RNase P (RP) gene transcript fragment (*RP*). Complete methods have been reported elsewhere^18^. Test results typically were available at 1 PM on the same day as when they were collected.

### Calculation of SARS-CoV-2 doubling time

The in vivo SARS-CoV-2 doubling time (*T*_*d*_) during the exponential growth phase (i.e., proliferation phase) was calculated from the corresponding rate constant (*k*) according to Eq. 1 and Eq. 2:

1 $${{{{\mathrm{ln}}}}}(y)=\,{{{{\mathrm{ln}}}}}({y}_{0})+k\cdot t$$

2 $${T}_{d}=\frac{{{{{\mathrm{ln}}}}}(2)}{k}$$

where, *y* is the SARS-CoV-2 RNA copy number per swab; *y*_*0*_ is the initial SARS-CoV-2 RNA copy number per swab; and *t* is time.

### Model fitting of the temporal SARS-CoV-2 concentration trajectories

The employed model generally was based on the framework described by Kissler et al.^8^ (available at: https://github.com/gradlab/CtTrajectories). The model used viral load concentration-time data, using the cycle threshold (*C*_*t*_) values measured by RT-qPCR. The *C*_*t*_ value represents the number of thermal cycles needed to amplify the viral RNA, following transcription into complementary DNA (cDNA), to a detectable signal. Since we only had one group in our analysis pipeline, we did not use the hierarchical structure component (i.e., Variant *versus* NonVariant). We removed sequences of three, or more, consecutive negative test results (*C*_*t*_ = 40) to avoid overfitting to these trivial values. For the main analysis, prior information was used from a previous analysis^8^. We also conducted a sensitivity analysis using vague priors as well as a strongly biased set of priors to assess robustness to the choice of prior. The settings for these priors are presented above in the Results section.

### Plasma cytokine concentration analysis

The concentration of 21 inflammatory markers in cryopreserved plasma samples was measured using the MILLIPLEX® human cytokine, chemokine, and growth factor panel (HCYTA-60K-21C, EMD Millipore, Burlington, MA) bead-based multiplex assay on a MAGPIX® instrument (EMD Millipore) according to the manufacturer’s instructions. The analytes were: soluble CD40L (sCD40L), granulocyte-macrophage colony-stimulating factor (GM-CSF), interferon alpha-2 (IFN-α2), interferon gamma (IFN-γ), interleukin-1 alpha (IL-1α), interleukin-1 beta (IL-1β), interleukin-1 receptor antagonist (IL-1Ra), interleukin-2, −4, −6, −8, −10, −12 p70, −13, −15, −17A (IL-2, IL-4, IL-6, IL-8, IL-10, IL-12 p70, IL-13, IL-15, IL-17A), interferon γ-induced protein-10 (IP-10), monocyte chemoattractant protein-1 (MCP-1), macrophage inflammatory protein-1 alpha (MIP-1α), macrophage inflammatory protein-1 beta (MIP-1β), and tumor necrosis factor alpha (TNF-α). Measurements below the lower limit of quantification were not reported.

### Quantification of serum IgG, IgM, and IgA against SARS-CoV-2

Measurement of serum anti-receptor-binding domain (RBD) IgG, IgM, and IgA concentrations was carried out using an enzyme-linked immunoassay (ELISA) using methods described in detail elsewhere^18,19^. Briefly, 96- well microtiter plates were coated with 2 µg mL^−1^ recombinant RBD protein in calcium- and magnesium-free phosphate-buffered saline (PBS), followed by triple-washing with PBS containing 0.1% Tween-20 (TPBS), and incubation with PBS containing 3% dried milk (Bioworld, Dublin, OH) 1 h at room temperature (RT). Participant serum was added in duplicate serial dilutions, incubated for 2 h at RT, and washed three times with TPBS. Bound antibodies were detected using goat anti-human IgG, IgM, or IgA conjugated with horseradish peroxidase (Bethyl Laboratories, Montgomery, TX), added in PBS at 1:50,000 at RT for 1 h. After three washes with TPBS, tetramethylbenzidine substrate solution (100 µL, ThermoFisher Scientific, Waltham, MA) was added for 10 min at RT followed by sulfuric acid stop solution (100 µL, ThermoFisher Scientific, Waltham, MA) for light absorption measurements at 450 and 650 nm (Spark 10 M, Tecan, Baldwin Park, CA). Each plate contained a control titration of the anti-RBD monoclonal antibody CR3022 in IgG, IgM, or IgA format (Creative Biolabs, Shirley, NY) to provide a standard curve. Serum anti-RBD IgG binding activity was expressed as an equivalent to a concentration of CR3022. The lower detection limit was *ca*. 3 ng mL^−1^ control antibody (*ca*. 100 ng mL^−1^ for diluted serum).

### IFN-γ ELISpot assay for CD8^+^ T-cell responses

These assays were performed as previously described^20^. Thawed cryopreserved PBMC were plated at 1–2 million cells/well in RPMI with IL-2 at 50 U mL^−1^ (NIH AIDS Reagent Repository Program) with a CD3:CD4 bi-specific monoclonal antibody (gift of Dr. J Wong) and cultured for *ca*. 14 days to yield purified polyclonal CD8^+^ T cells. These cells were viably cryopreserved until the day of ELISpot assay.

Cells were added to a 96-well filter plate that had been precoated with an anti-IFN-γ antibody (Mabtech, Nacka Strand, Sweden) with the addition of a peptide pool, medium alone (three wells), or medium with PHA (Sigma Aldrich, St. Louis, MO) at 25 μg mL^−1^. After overnight incubation in a humidified CO_2_ incubator, the plate was washed and stained with biotinylated anti-IFN-γ antibody (Mabtech, Nacka Strand, Sweden) for visualization using a streptavidin-peroxidase reagent and counting on an automated ELISpot reader (AID, Autoimmun Diagnostika GMBH, Strassberg, Germany) against synthetic overlapping peptide pools spanning SARS-CoV-2 spike, nucleocapsid, matrix, and envelope proteins (NR-52402, NR-52404, NR-52403, NR-52405, BEI Resources, Manassas, VA). The response against each peptide pool was expressed as the raw count minus the mean of the triplicate negative control wells. The two following criteria needed to be met for positivity: ≥50 SFC/10^6^ CD8^+^ T lymphocytes and ≥ mean and two standard deviations of negative control wells (no peptide).

### Statistics and reproducibility

Datasets were analyzed using GraphPad Prism (version 9.3.1; GraphPad Software, Inc., La Jolla, CA). Serum IgA and IgM concentration half-lives (*t*_*1/2*_) were compared using a Wilcoxon matched-pairs signed rank test (paired *t*-test; nonparametric) and a Mann–Whitney test (unpaired rank test; nonparametric).

Reproducibility of the experiments, including sample sizes as well as the number and nature of replicates, were as follows. Clinical swab specimens collected from study participants were analyzed individually for SARS-CoV-2 RNA by RT-qPCR. Three target sequences (two viral, one host) were amplified simultaneously in a 96-well format, and each plate also included one human specimen control as well as one positive control. Plasma cytokine concentration measurements were carried out using two replicates consisting of paired aliquots from the same clinical sample. Each 96-well plate also contained two quality controls and seven standards, all in duplicate. Serum antibody measurements by ELISA were carried out on individual samples, run in four serial four-fold dilutions, and OD values were compared against a standard curve with the control antibody CR3022 in serial three-fold dilutions on the same 96-well plate. Cryopreserved PBMC samples were analyzed individually for CD8^+^ T-cell responses using an ELISpot assay. Each sample was run in a single well on a 96-well plate that also included triplicate negative control wells and duplicate positive control wells. The mean of the negative control wells was subtracted from the value of each sample well.

### Reporting summary

Further information on research design is available in the Nature Research Reporting Summary linked to this article.

## Results

### Update on ongoing workplace SARS-CoV-2 surveillance clinical study

On March 23, 2020, Oak Crest initiated the ongoing clinical study entitled, “Longitudinal Characterization of COVID-19 Prevalence and Incidence in a Small Working Institution with Both Public Health and Diagnostic Aims”^18^. The two primary study aims are to: (i) characterize the rate of SARS-CoV-2 acquisition in a small cohort of participants interacting on a daily basis in the workplace; and (ii) determine the ability of these data to manage workplace SARS-CoV-2 exposure and consequences, minimizing further spread as per public health advisories. In addition, related exploratory study goals include characterizing the rate of SARS-CoV-2 acquisition in employee and household members, quantifying antibody-specific responses in blood at baseline (previously exposed) and while on the study (to capture asymptomatic/presymptomatic, newly infected individuals), and characterizing viral shedding parameters in saliva and stool samples. The study has remained active to date, and sampling of participants has continued three times per week, except for a period between June 9, 2021 to January 3, 2022 where the testing frequency was reduced to twice per week due to low SARS-CoV-2 positivity rates in Los Angeles County. To date, 142 participants have been enrolled, and close to 7000 SARS-CoV-2 RT-qPCR tests (21,000 reactions) have been performed.

The high incidence in Los Angeles County COVID-19 cases between mid-November, 2020 and mid-March, 2021—peak 7-day positivity rate for Los Angeles County over this period was 17.7% on Dec. 26, 2020^21^—was reflected in positive RT-qPCR SARS-CoV-2 test results for nine study participants (Table 1). All were unvaccinated against SARS-CoV-2 at that time, and none had reported a previous case of symptomatic COVID-19 or a positive qPCR SARS-CoV-2 test result.

**Table 1:** Demographics of study participants testing positive for SARS-CoV-2 by RT-qPCR who were followed longitudinally.

Characteristic	Value
No. of participants	9
Female, no. (%)	6 (67)
Male, no. (%)	3 (33)
Age (yrs), median (range)	25 (19–53)
Race and ethnicity, no. (%)	
Black or African-American	0
White	9 (100)
Hispanic	6 (67)
Non-Hispanic	3 (33)
Asian	0
Other	0

All participants were unvaccinated against SARS-CoV-2 at the time of the first positive test result.

Employees who tested positive immediately were isolated, and, consequently, no workplace SARS-CoV-2 transmissions occurred. Four individuals (subjects 2, 21, 38, and 48) from this positive cohort of nine individuals (Table 1, subjects 2, 21, 38, 48, 63, 82, 83, 84, and 85) were available for direct observation and frequent sampling during the early phase of infection (i.e., proliferation phase). At some point post-diagnosis, all four subjects reported symptoms of headaches, body aches, chills, sore throat, cough, runny nose, nausea, diarrhea, vomiting, and/or nasal drip; each subject reported three or more symptoms. These symptoms were consistent with those reported by the remaining four individuals from the positive cohort, with one asymptomatic individual (subject 82). No participants were hospitalized. The duration of participants’ identified symptoms ranged from 10 days to >2 months. During the above period, intensive longitudinal sample collection was performed for participants testing positive for SARS-CoV-2, and the corresponding results are presented below. No association between participant symptomology and SARS-CoV-2 viral load or host responses was observed.

### Transmission electron microscopy (TEM) of clinical nasal swab specimens reveal tightly bound cells and a complex microenvironment

**Fig. 1:**
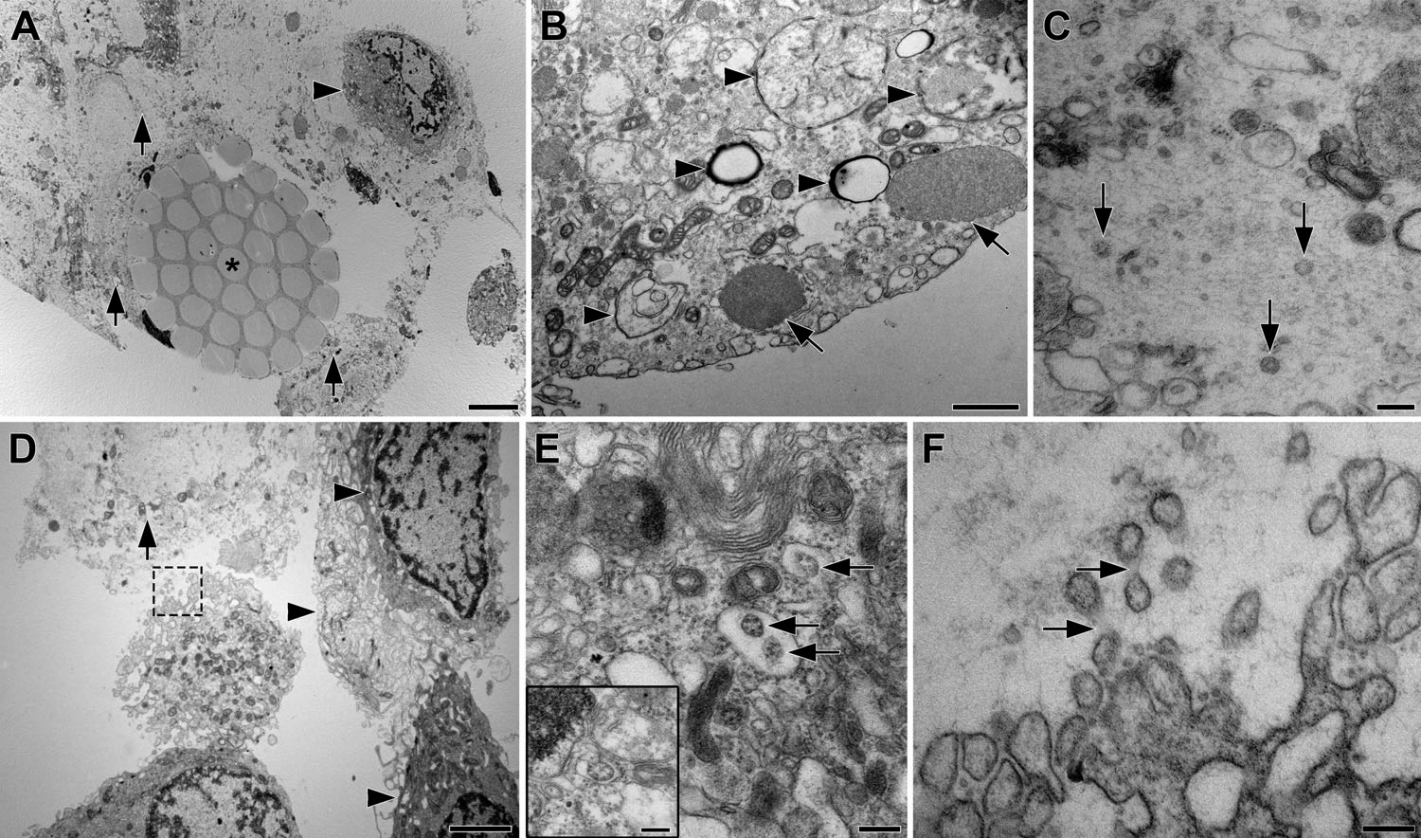
TEM Images of clinical nasal swab specimens collected from a participant (subject 2), who tested positive for SARS-CoV-2 RNA by RT-qPCR. **A** Cross-section through a swab fiber bundle (asterisk) with lysed (arrows) and intact cells (arrowheads); scale bar, 5 µm. **B** Cytoplasm of an intact epithelial cell with possible viral double membrane assembly structures (arrowheads) and cytoplasmic aggregates of unknown origin (arrows); scale bar, 1 µm. **C** Extracellular space with cytoplasmic material from lysed cells and three possible SARS-CoV-2 virions (arrows); scale bar, 200 nm. **D** Layer of intact epithelial cells with complex, interdigitating membrane protrusions (arrowheads) and lysed cell (arrow); scale bar, 2 µm. The cell in the center extends membrane protrusions into the lysed material outlined with a dashed box, shown at higher magnification in **F**. **E** Cytoplasm of an epithelial cell showing “outside-in” ribosomal structures (arrows)^22^ that are easily mistaken for virions, but are generated by budding of rER membranes into the lumen (insert); scale bar, 200 nm. **F** Plasma membrane of the boxed cell in **D** with convoluted membrane protrusions that are easily mistaken for virions, but can be identified as protrusions by faint connecting densities (arrows); scale bar, 200 nm.

Nasal swabs (25-086-PD, Puritan Medical Products, Guilford, ME) from study participants found to be positive for SARS-CoV-2 RNA by RT-qPCR were examined by TEM. Representative images are shown in Fig. 1, demonstrating the close association of cells with swab fibers and multiple ultrastructural features that complicate the identification of SARS-CoV-2 virions.

The majority of cells directly attached to swab fibers were lysed and adsorbed between individual fibrils. The lysed cells commonly were surrounded by a second layer of attached intact cells, with little extracellular material only present between the cells (Fig. 1A). Within the entire cell population, only a small subset showed signs of viral infection evidenced by the presence of unusual double membrane structures that were not observed in epithelial cells of an RT-qPCR-negative specimen, suggestive of viral assembly organelles (Fig. 1B). We observed three possible SARS-CoV-2 virions in the extracellular space that displayed virion features and were of the appropriate diameter (99, 99, and 92 nm, Fig. 1C). However, unambiguous structural identification of virions was not possible due to the presence of similar cellular features (e.g., vesicles in the cytoplasm). When the material was present in the extracellular space, it consisted mostly of cytoplasm released from adjacent lysed cells (Fig. 1D). Furthermore, the different cell types also formed confusing cellular structures such as vesicles with “outside-in” ribosomes and extracellular protrusions (Fig. 1E, F).

### SARS-CoV-2 viral kinetics differ across anatomic compartments

**Fig. 2:**
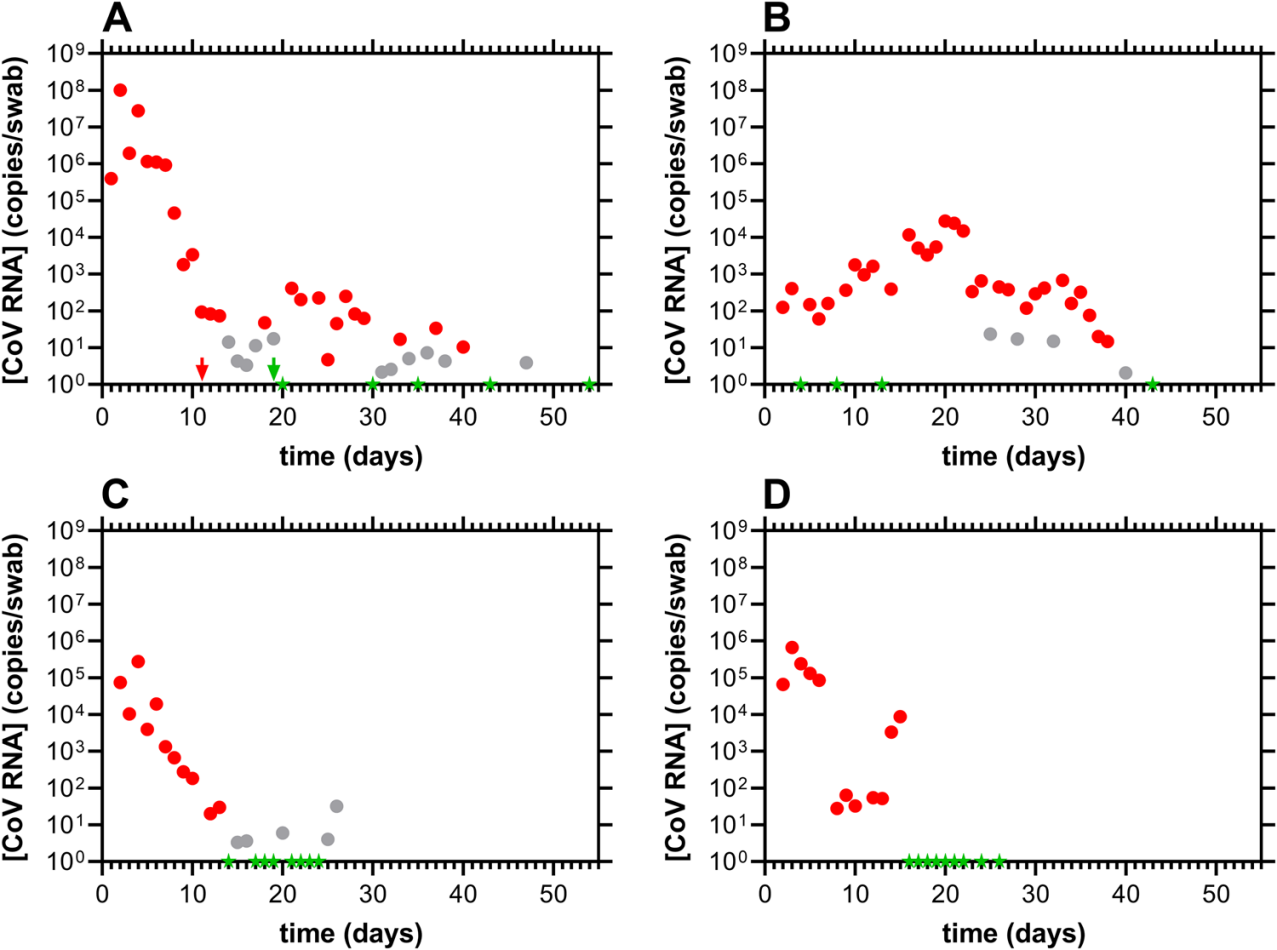
Longitudinal viral load kinetics for subject 21 across multiple anatomic compartments. Red, positive; gray, inconclusive (i.e., only one of the two oligonucleotide probes targeting the viral nucleocapsid protein gene transcript fragment met the assay threshold for positivity); green, negative (i.e., both viral probes led to *C*_*t*_ values above 40, but the human gene transcript control had a *C*_*t*_ value below 40. These consist of valid samples where the SARS-CoV-2 content was below the limit of quantitation of the assay); arrows designate clinical RT-qPCR test results (i.e., from separate, CLIA laboratory, outside of current clinical study); the axis ranges are the same across all four panels for ease of comparison. **A** nasal swab viral RNA copy dynamics; **B** stool swab viral RNA copy dynamics; **C** oral swab viral RNA copy dynamics; **D** viral RNA copy dynamics in saliva samples processed with the Super SAL2 kit (Oasis Diagnostics, Vancouver, WA). The qPCR assay, and the associated interpretation of test results, have been discussed in detail elsewhere^18^.

One participant (subject 21) provided nasal, oral, and stool samples for up to 54 days. The SARS-CoV-2 copies per swab, based on RT-qPCR analyses of the samples in all three compartments, are shown in Fig. 2.

The viral load kinetic profiles across compartments were heterogeneous and decayed rapidly in nasal and oral swab samples (Fig. 2A, C). However, the maximal SARS-CoV-2 copies (*C*_*max*_) were 370 times higher in the nasal swab samples [*C*_*max*_(nasal), 1.01 × 10^8^ copies/swab; *C*_*max*_(oral), 2.75 × 10^5^ copies/swab]. Oral swab and saliva samples collected and pre-purified with the Super SAL2 kit collection device afforded similar SARS-CoV-2 copy numbers (Fig. 2C, D) and decay profiles, but the swab data were less noisy. The SARS-CoV-2 dynamics in stool specimens (Fig. 2B) were different from the other sampled compartments. A bimodal profile was observed and the *C*_*max*_ was 3800 times lower than in the nasal swab samples [*C*_*max*_(stool), 2.66 × 10^4^ copies/swab], while the time to reach *C*_*max*_ (*t*_*max*_) was 18 days later [*t*_*max*_(nasal), 2 d; *t*_*max*_(stool), 20 d]. Despite this large difference in *C*_*max*_, subject 21 remained positive for SARS-CoV-2 RNA for close to 40 days in nasal and stool samples, while positivity in oral samples only lasted for *ca*. two weeks.

### Temporal SARS-CoV-2 profiles are heterogeneous

**Fig. 3:**
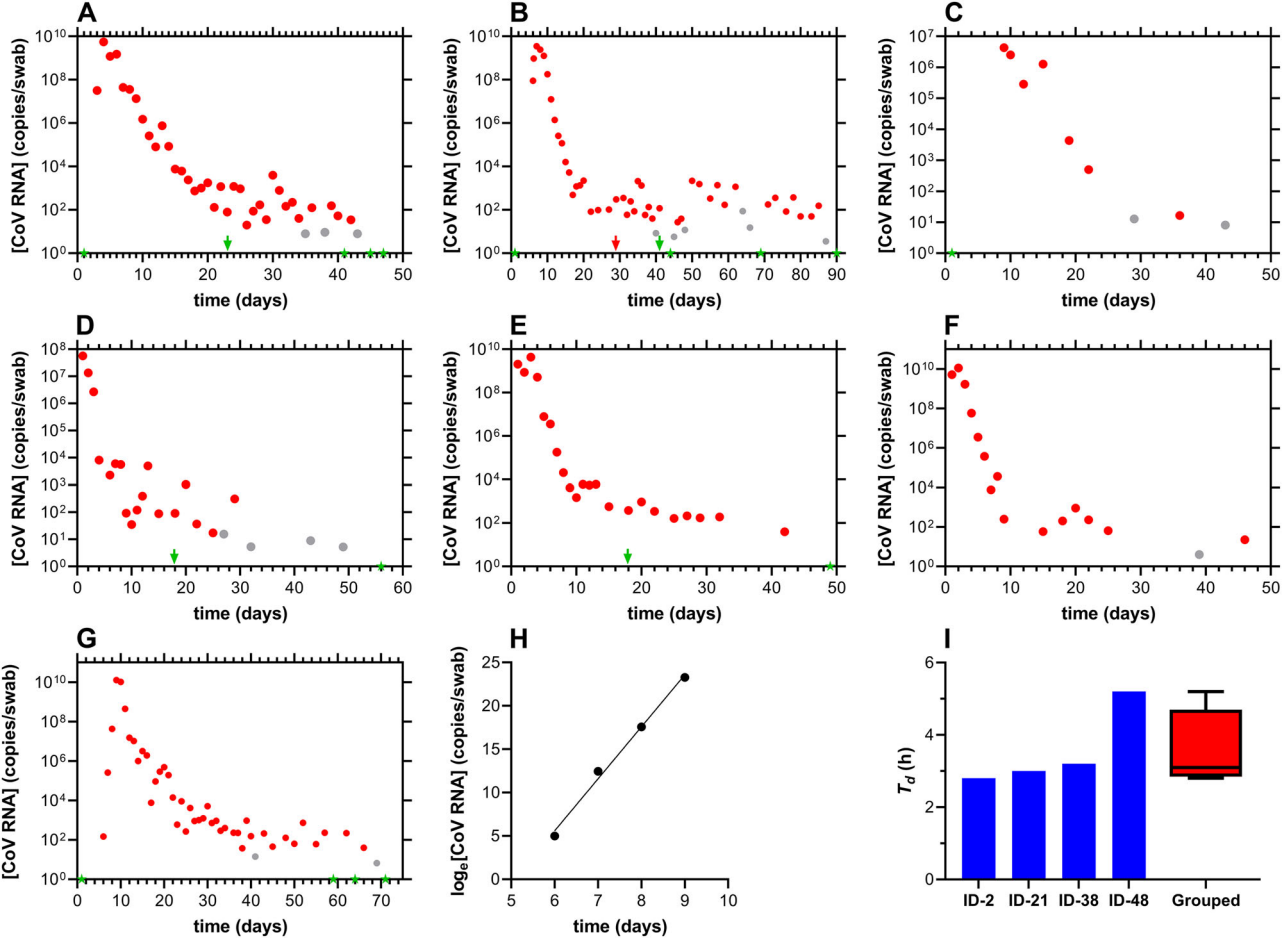
Longitudinal nasal swab SARS-CoV-2 viral load kinetics. Red, positive; gray, inconclusive; green, negative; arrows designate clinical qPCR test results (i.e., from separate, CLIA laboratory, outside of current clinical study). **A** subject 38; **B** subject 48; **C** subject 63; **D** subject 83; **E** subject 84; **F** subject 85; **G** subject 2, where the complete growth curve life-cycle is captured, including short lag phase between a negative test result (green star) and the first positive test result (red circle), a rapid exponential growth phase, and a slow decline phase. **H** Plot of exponential growth phase for subject 2 used to estimate a SARS-CoV-2 in vivo doubling time of 2.8 h (*R*^2^ = 0.9934). **I** In vivo SARS-CoV-2 doubling times during the exponential growth phase for four subjects; subject 2, 2.8 h; subject 21, 3.0 h; subject 38, 3.2 h; subject 48, 5.2 h; grouped, box plot of doubling times, *T*_*d*_, for all four study participants. The box extends from the 25th to 75th percentiles, with the horizontal line in the box representing the median (3.1 h); whiskers represent the lowest and highest datum.

Seven study participants were followed longitudinally as their SARS-CoV-2 RT-qPCR status changed from negative to positive. The nasal swab viral copy numbers as a function of time are shown in Fig. 3 (see Supplementary Data 1 for the complete source dataset).

The overall features of the SARS-CoV-2 RNA concentration-time semilog plots are shared across eight participants (Figs. 2A and 3) and follow similar trends. A rapid exponential growth (proliferation) phase is followed by a bimodal decay in SARS-CoV-2 RNA copies, characterized by an initial rapid decay followed by a slow, second decay (clearance) phase. The length of the clearance profile varied widely across participants and, in several cases, the decline in RNA copy numbers was interrupted by small concentration spikes (e.g., Fig. 3F, Day 20), suggestive of multimodal decay kinetics. The extent of nasal viral replication at later timepoints in the clearance phase is unknown, as is the associated potential for infectivity. However, the observed viral load maxima in the 10^3^–10^4^ copies per swab range (e.g., Fig. 2A, B, F) 3 weeks, or later, after the first positive test result suggests that viral replication may still be ongoing.

The highest measured viral loads for each participant, *C*_*max*_(CoV RNA), varied across multiple orders of magnitude and were: subject 21 (Fig. 2A), 1.0 × 10^8^ copies/swab; subject 38 (Fig. 3A), 5.5 × 10^9^ copies/swab; subject 48 (Fig. 3B), 3.5 × 10^9^ copies/swab; subject 63 (Fig. 3C), 4.6 × 10^6^ copies/swab; subject 83 (Fig. 3D), 5.7 × 10^7^ copies/swab; subject 84 (Fig. 3E), 4.3 × 10^9^ copies/swab; subject 85 (Fig. 3F), 1.1 × 10^10^ copies/swab; subject 2 (Fig. 3G), 1.3 × 10^10^ copies/swab.

Some of the study participants obtained complementary, nasopharyngeal RT-qPCR SARS-CoV-2 tests through CLIA laboratories (arrows in Figs. 2A, 3A, B, D, E). It is noteworthy that in four out of seven tests (57%), a negative result was obtained with the CLIA test, while a positive test result was obtained in our study on the same sampling day. Differences in the initial stages of sample preparation in the two tests (i.e., the current study and CLIA laboratory) present potential consequences for assay sensitivity. In our study, swab samples were either collected dry (i.e., no preservative added, processed within *ca*. 40 min of collection) or preserved with RNA Shield (300 µL, Zymo Research, Tustin, CA) and typically processed within 24 h of collection. Extraction of viral RNA from these samples was carried out by first adding lysis buffer (400 µL) followed by column purification of the entire sample volume (i.e., either 400 or 700 µL). In CLIA laboratory SARS-CoV-2 RNA RT-qPCR tests, the swab is preserved in larger volumes of transport media (typically 3–10 mL), and in the subsequent RNA extraction step, an aliquot of the sample is used. It is therefore expected that, for the same sample, lower quantities of viral RNA are analyzed in the CLIA laboratory test than in our study, likely leading to lower sensitivities for the former. These considerations need to be taken into account when comparing cycle threshold (*C*_*t*_) values, or viral RNA copies per unit volume, across studies. Consequently, we have reported viral loads as SARS-CoV-2 copies per swab above.

### SARS-CoV-2 Doubling times in the growth phase generally are remarkably consistent

The regular, repeated, high-intensity sampling in our observational study allowed early identification of SARS-CoV-2 positivity, providing an opportunity for measuring the in vivo SARS-CoV-2 doubling time, *T*_*d*_, during the growth phase. Successful mapping of the growth phase is best described by Fig. 3G, H. The slope of the semilog plot shown in Fig. 3H was used to calculate *T*_*d*_ for subject 2 (2.8 h), and a similar approach was employed for three additional participants (subjects 21, 38, and 48) where sufficient early-stage data were available (Fig. 3I). Three out of the four participants had remarkably similar doubling times, while one participant (subject 48) exhibited considerably slower SARS-CoV-2 growth (Fig. 3I). The *T*_*d*_ value for subject 48 was calculated from three timepoints spanning 24 h (Fig. 3B). Subject 48 also took longer to clear the virus than any of the other participants (Fig. 3B).

### Viral dynamics model analysis

A Bayesian model^8^ was used to estimate individuals’ peak viremia, and duration of the viral proliferation and clearance phases based on the experimental data shown above. We removed a series of three or more consecutive negative tests (*C*_*t*_ = 40) to avoid overfitting these trivial values. Viral load was log_10_-transformed, and a piece-wise linear model was fitted using control points for time of infection, time and height of peak viral load, and time to infection clearance. The control points were inferred with the Hamiltonian Monte Carlo method using Stan (version 2.21.0) in *R* studio (version 1.2.5033)^23^.

For the main analysis we used the priors (informative, uninformative, and biased set) as described in Kissler et al.^8,24^. The term “informative priors” literally refers to “prior beliefs”; i.e., what was known before the experiment. The priors also can be uninformed, also known as “uniform”, where no assumptions from prior experiments are made, or strongly biased. We also conducted a sensitivity analysis using all three approaches to assess the robustness of the choice of priors (vide infra).

Informative prior settings were used as in Kissler et al.^8^:

*μ*_*ωρ*_ ~ Normal(2.7, 14/6) [0.25, 14]

*μ*_*ωr*_ ~ Normal(7.4, 30/6) [2,30]

*μ*_*δ*_ ~ Normal(20, 40/6) [0, 40]

**Fig. 4:**
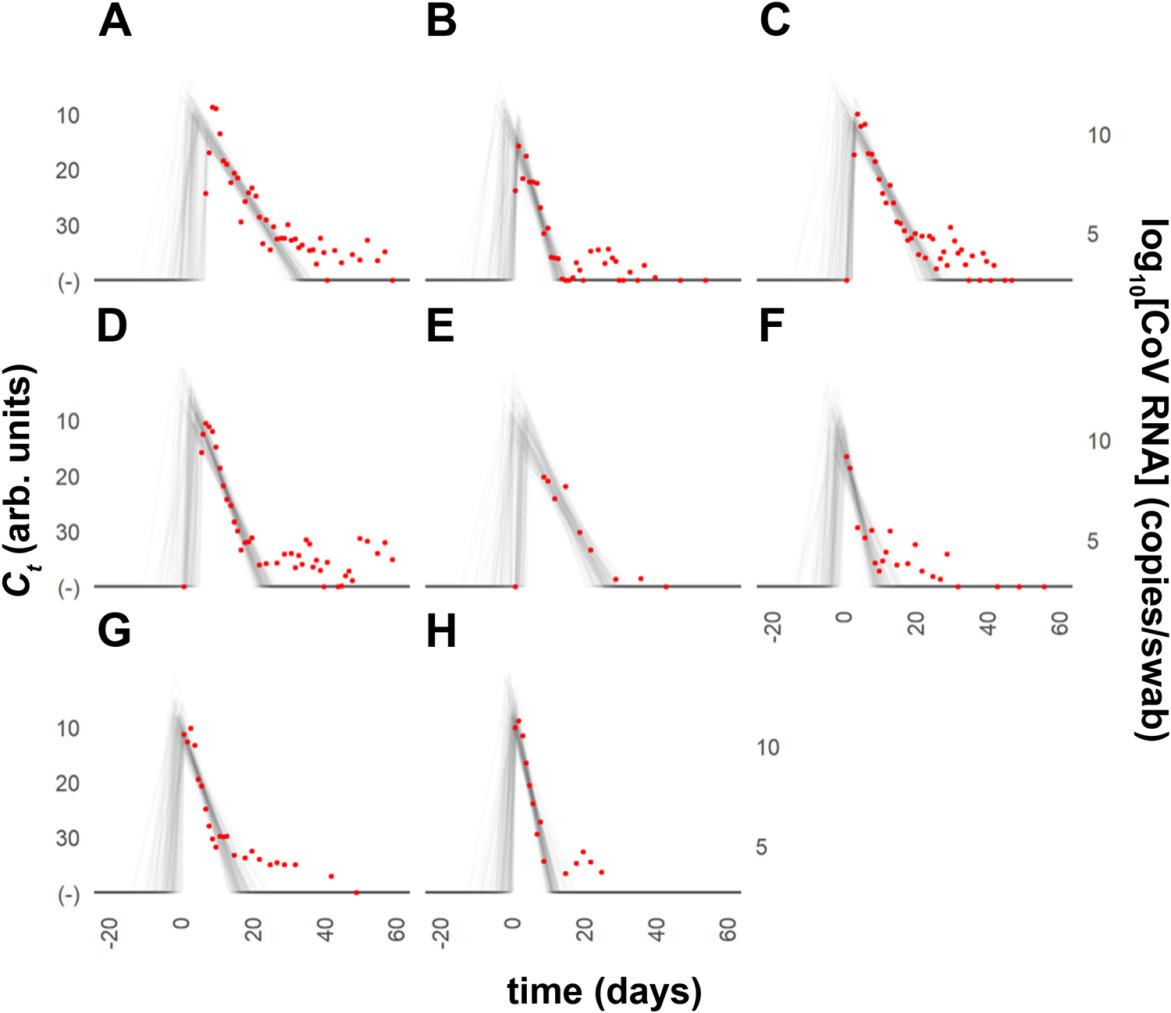
Modeled nasal swab SARS-CoV-2 *C*_*t*_ trajectories for individual study participants. Informative priors model plots (gray) for *C*_*t*_ values and estimated trajectories for infections; each red circle corresponds to one observation; T (d) indicates the time since the minimum *C*_*t*_ value (highest viral load). **A** subject 2; **B** subject 21; **C** subject 38; **D** subject 48; **E** subject 63; **F** subject 83; **G** subject 84; **H** subject 85.

Where *ω*_*p*_ is the duration of the proliferation stage (constrained to 0.25–14 days), *ω*_*r*_ is the duration of the clearance stage (constrained to 2–30 days to prevent inferring unrealistic values), *δ* (constrained to 0–40) is the absolute difference in *C*_*t*_ between the limit of the detection and the lowest *C*_*t*_. The estimated trajectory using the informative priors for eight individuals is shown in Fig. 4.

For sensitivity analysis, we conducted a similar analysis (see Supplementary Fig. 1 in the Supplementary Information) with the following set of uninformative priors settings:

*μ*_*ωρ*_ ~ Normal(14/2, 14/6) [0.25, 14]

*μ*_*ωr*_ ~ Normal(30/2, 30/6) [2,30]

*μ*_*δ*_ ~ Normal(40/2, 40/6) [0, 40]

We then applied the model using the following set of biased prior settings (see Supplementary Fig. 2 in the Supplementary Information):

*μ*_*ωρ*_ ~ Normal(0, 14/6) [0.25, 14]

*μ*_*ωr*_ ~ Normal(0, 30/6) [2,30]

*μ*_*δ*_ ~ Normal(20, 40/6) [0, 40]

The same parameter constraints were used for each set of priors. Viral trajectories estimated for different priors are compared in Table 2.

**Table 2:** Estimated viral trajectories for different priors presented as means with the 95% confidence interval (CI) in brackets.

	Informative priors	Uninformative priors	Biased priors
Clearance time (d)	15.64 (13.09, 18.81)	17.16 (14.54, 21.29)	15.25 (12.73, 16.00)
Peak *C*_*t*_ value	10.49 (6.93, 14.80)	10.43 (6.79, 15.12)	10.52 (7.02, 14.93)
Proliferation time (d)	3.07 (0.68, 7.08)	5.95 (1.42, 9.91)	2.42 (0.54, 6.91)

Table 2 shows the results of the model analysis under three sets of different priors. The estimated values are fairly consistent under each prior, but it should be noted that our sample size was limited. We observed a longer mean clearance time than reported by Kissler et al.^8^, which could be due to our extended and more frequent sampling period. Further, the mean peak *C*_*t*_ value was much lower in our sampled population (i.e., higher viral loads) compared to the reference study^8^.

### Kinetics of innate immune activation and responses differ among participants

**Fig. 5:**
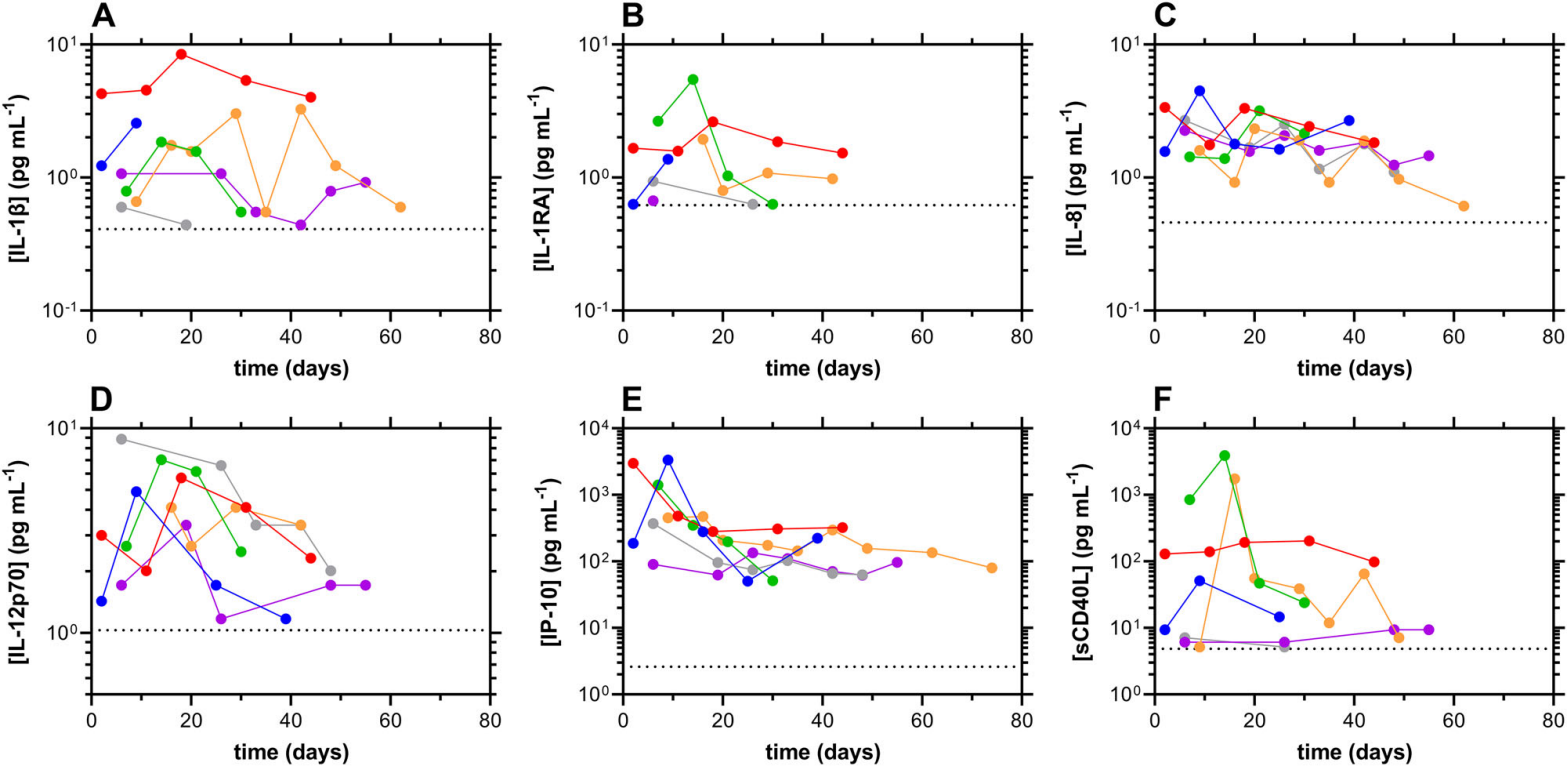
Cytokine/chemokine concentration-time profiles for participants testing positive nasally for SARS-CoV-2 RNA, normalized temporally to the first day of positivity by RT-qPCR. Not all subjects testing positive participated in the blood collection portion of the study. The dotted line represents the assay lower limit of quantitation; blue, subject 2; red, subject 21; green, subject 38; orange, subject 48; magenta, subject 83; gray, subject 84. **A** IL-1β; **B** IL-1RA; **C** IL-8; **D** IL-12 p70; **E** IP-10; **F** sCD40L.

Longitudinal plasma cytokine concentrations for participants testing positive for SARS-CoV-2 are shown in Fig. 5. The data are normalized temporally to the day of the first positive SARS-CoV-2 RT-qPCR test result (Day 1). Additionally, related cytokine data are included in Supplementary Fig. 3 in the Supplementary Information, and the complete source dataset is included in Supplementary Data 2.

Overall, the cytokine/chemokine concentrations declined from the first day of SARS-CoV-2 RT-qPCR positivity, but the temporal profiles varied across participants and cytokines. Interleukin-1 beta (IL-1β) and interleukin-1 receptor antagonist (IL-1Ra) plasma concentration timeseries did not follow a consistent trend across participants but appeared to peak around Day 20 prior to declining for two individuals (subjects 21 and 38, Fig. 5A, B). Interleukin-8 and -12 plasma concentrations generally declined over time for all participants (Fig. 5C, D). Concentrations of the chemokine interferon gamma-inducible protein-10 (IP-10, also known as CXCL10) generally decreased slowly over time, except in subject 83 where they remained constant (Fig. 5E). The most disparate temporal concentration profiles for the measured inflammatory markers were observed for soluble CD40L (sCD40L, Fig. 5F), shed by activated T lymphocytes and platelets. In two participants (subjects 21 and 83), the plasma concentrations remained relatively constant over 60 days. In two other participants (subjects 38 and 48), the concentrations rose sharply within the first three weeks and then declined rapidly. The final two participants (subjects 2 and 84, barely visible behind the plot from subject 83) only had quantifiable concentrations in the first 25 days.

There were no notable temporal changes in the plasma concentrations of the remaining inflammatory markers measured (Supplementary Fig. 3). Human interferon alpha-2 (IFNα2) and interferon gamma (IFNγ) only were detected in a small subset of samples. Interleukin-1 alpha (IL-1α) and interleukin-2, −4, −6, −10, −13, −15, and −17A (IL-2, IL-4, IL-6, IL-10, IL-13, IL-15, and IL-17A) concentrations either were below the limit of quantification in most samples (IL-2, IL-6, and IL-15), or were only observed in a minor subset of samples. Monocyte chemoattractant protein-1 (MCP-1) and macrophage inflammatory protein-1 alpha and beta (MIP-1α and MIP-1β) plasma concentrations remained constant over time in the majority of samples. The concentration of tumor necrosis factor alpha (TNF-α) was not quantifiable in most samples except for subject 48, where a concentration maximum was observed at Day 16, followed by a concentration plateau until the last sample on Day 74 (Supplementary Fig. 3).

### Humoral immune response temporal decay profiles differ widely across participants

**Fig. 6:**
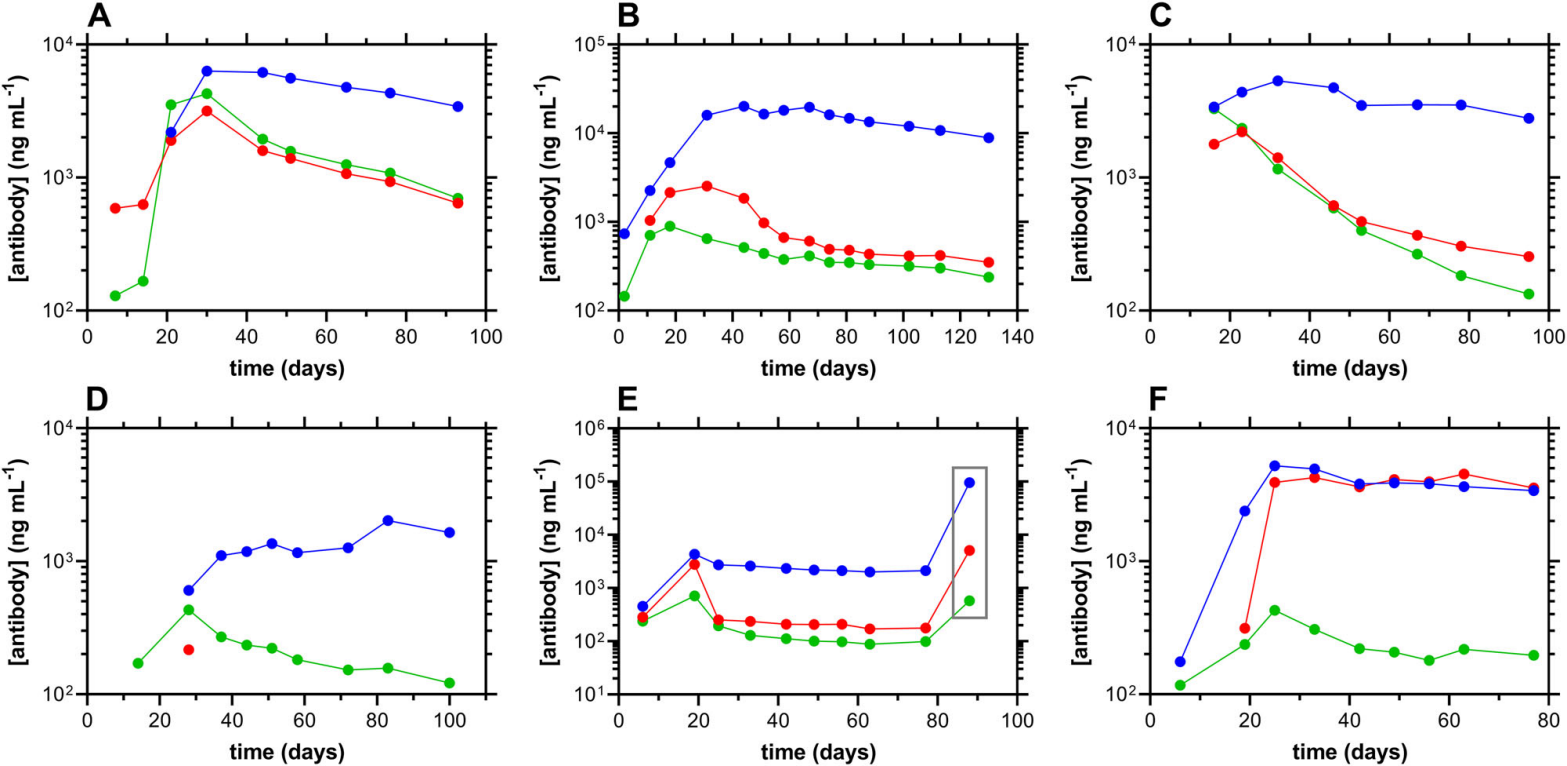
Longitudinal antibody responses to SARS-CoV-2 in serum samples from six study participants, normalized temporally to the first day of positivity by RT-qPCR. Blue, IgG; red, IgA; green, IgM. **A** subject 2; **B** subject 21; **C** subject 38; **D** subject 48; **E** subject 83, with gray box identifying timepoint post-immunization with Ad26.COV2.S vaccine (Research name: JNJ-78436735, Janssen Pharmaceutical Companies, also known as the Johnson and Johnson vaccine); **F** subject 84.

Longitudinal humoral responses against SARS-CoV-2 in the study cohort are shown in Fig. 6 (see Supplementary Data 3 for the complete source dataset). While the IgG concentrations generally were the most stable, the temporal decay profiles are strikingly different across participants. Robust responses were measured for all three antibodies (IgG, IgA, and IgM) over the *ca*. 3-month period following the first SARS-CoV-2 positive RT-qPCR test result, with the exception of subject 48 (Fig. 6D). For this participant, IgG responses were lower than for the rest of the cohort and were delayed. IgA Concentrations were only quantifiable at one timepoint (Day 28).

When antibody concentrations declined over time, the corresponding half-lives, *t*_*1/2*_, could be calculated using a one-phase decay model. Due to the stability of the IgG serum concentrations over the *ca*. 90-day window of analysis, only *t*_*1/2*_ values for IgA and IgM could be calculated for five individuals: *t*_*1/2*_(IgA): median, 8.8 d; min, 2.3 d; max, 32.1 d; *t*_*1/2*_(IgM): median, 10.4 d; min, 1.1 d; max, 10.7 d. When the IgA and IgM *t*_*1/2*_ values were compared using a Wilcoxon matched-pairs signed rank test, the datasets were found not to be significantly different (*P* > 0.9999). When an unpaired analysis was performed on the data using a Mann–Whitney test, the same result was obtained (*P* > 0.9999).

### An unusually mild case of COVID-19

**Fig. 7:**
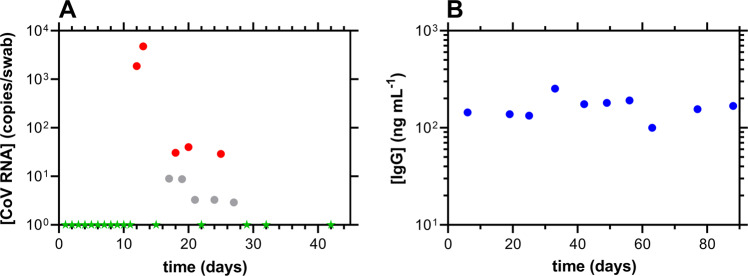
Longitudinal nasal swab SARS-CoV-2 viral load kinetics and corresponding humoral responses for subject 82. Day 1 corresponds to the first RT-qPCR sample. **A** nasal swab SARS-CoV-2 RT-qPCR measurements; red, positive; gray, inconclusive; green, negative. **B** Serum IgG measurements; IgA and IgM concentrations were BLQ in all serum samples.

One participant (subject 82) developed asymptomatic COVID-19, characterized by low SARS-CoV-2 viral loads in nasal swab samples [Fig. 7A; *C*_*max*_(CoV RNA), 4.8 × 10^3^ copies per swab]. The sudden shift to SARS-CoV-2 RT-qPCR positivity was preceded by eleven consecutive negative test results (green stars, Fig. 7A). Serum antibody concentrations over the same time period only could be quantified for IgG, and these remained stable throughout (Fig. 7B; median [IgG], 162 ng mL^−1^). Interestingly, the IgG measurement on Day 6 (144 ng mL^−1^) corresponds to the period of SARS-CoV-2 negativity by RT-qPCR.

### Cellular responses to possible SARS-CoV-2 exposures across the study cohort provided additional insights

**Fig. 8:**
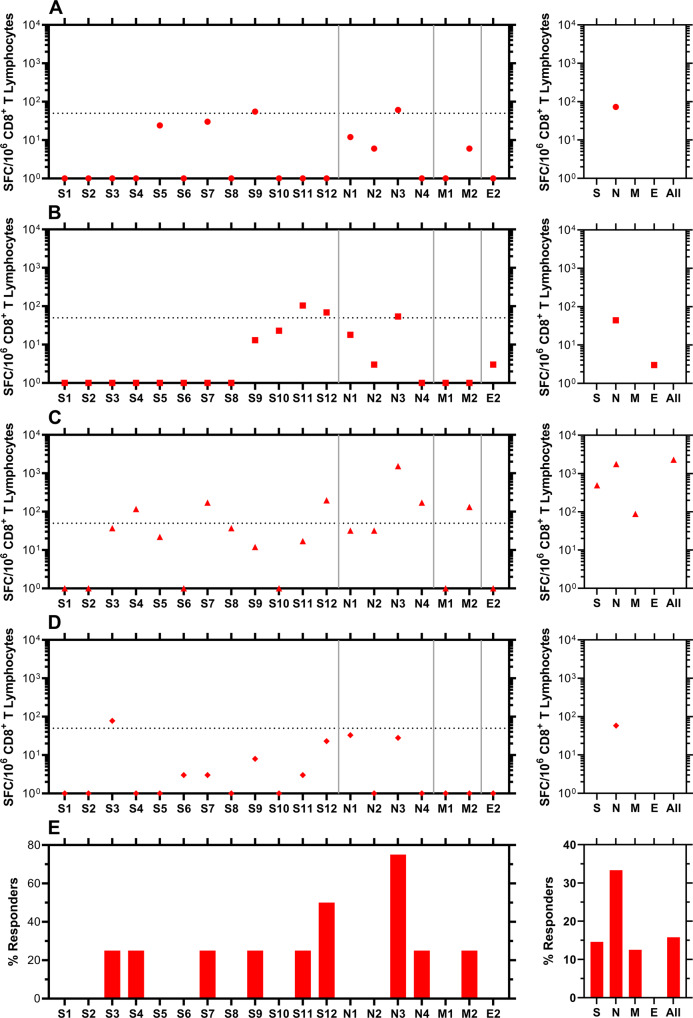
Evaluation of CD8^+^ T-cell targeting of SARS-CoV-2 in blood samples from participants who became positive for SARS-CoV-2 RNA by RT-qPCR 2–4 months earlier. IFN-γ ELISpot was performed on polyclonally expanded CD8^+^ T cells using peptides spanning spike, nucleocapsid, matrix, and envelope proteins that were combined in pools of 16 or fewer. Spike was contained in 12 pools (S1 to S12), nucleocapsid in four pools (N1 to N4), matrix in two pools (M1 to M2), and envelope in one pool (E2). Panels **A**–**D** present frequencies of responses from individual participants, while panel **E** provides summary data as percentages of persons responding against each pool. Each panel consists of two sub-panels, with the left sub-panel showing responses against individual peptides, while the right sub-panel shows the total responses for each peptide pool. Negative values for the responses were replaced by zeros in the left sub-panels. The total values for S, N, and M do not necessarily equal the sums of the pools because the sums were calculated including negative values after background subtraction. Horizontal dotted lines indicate the cutoff for positivity based on the following criteria: at least 50 SFC/10^6^ CD8^+^ T Lymphocytes and > mean and two standard deviations of negative control wells (no peptide). **A** Circles, subject 2; **B** squares, subject 21; **C** triangles, subject 38; **D** diamonds, subject 48.

Blood samples for analysis of possible SARS-CoV-2-targeted cellular immune responses were collected on April 1, 2021 from participants who contracted COVID-19 during our study (vide supra, Fig. 8) and others who remained RT-qPCR negative for SARS-CoV-2 RNA throughout (i.e., from March 23, 2020 to April 1, 2021, Fig. 9). The CD8^+^ T-cell responses were studied using IFN-γ ELISpot assays targeting pools of overlapping peptides spanning spike (12 pools), nucleocapsid (4 pools), matrix (2 pools), and envelope (1 pool) proteins. The complete source dataset is included in Supplementary Data 4.

**Fig. 9:**
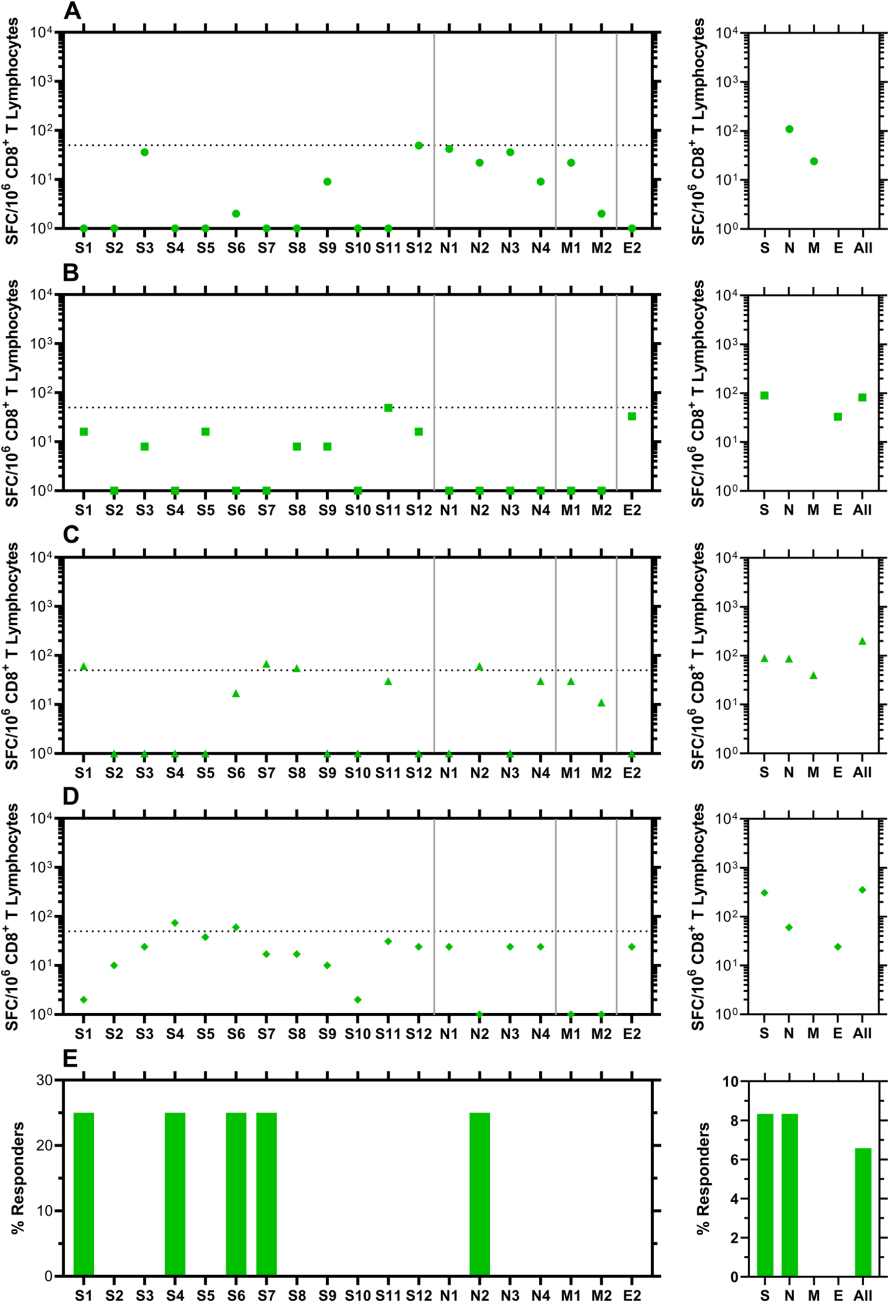
Evaluation of CD8^+^ T-cell targeting of SARS-CoV-2 in blood samples from participants who did not become positive for SARS-CoV-2 RNA by RT-qPCR between March 23, 2020 and April 1, 2021 (date of blood collection). IFN-γ ELISpot was performed on polyclonally expanded CD8^+^ T cells using peptides spanning spike, nucleocapsid, matrix, and envelope proteins that were combined in pools of 16 or fewer. Spike was contained in 12 pools (S1 to S12), nucleocapsid in four pools (N1 to N4), matrix in two pools (M1 to M2), and envelope in one pool (E2). Panels **A**–**D** present frequencies of responses from individual participants, while panel **E** provides summary data as percentages of persons responding against each pool. Each panel consists of two sub-panels, with the left sub-panel showing responses against individual peptides, while the right sub-panel shows the total responses for each peptide pool. Negative values for the responses were replaced by zeros in the left sub-panels. The total values for S, N, and M do not necessarily equal the sums of the pools because the sums were calculated including negative values after background subtraction. Horizontal dotted lines indicate the cutoff for positivity based on the following criteria: at least 50 SFC/10^6^ CD8^+^ T Lymphocytes and >mean and two standard deviations of negative control wells (no peptide). **A** Circles, subject 17; **B** squares, subject 18; **C** triangles, subject 24; **D** diamonds, subject 54.

The overall response patterns in Fig. 8 indicate the highest targeting of nucleocapsid protein regions, followed by similar targeting frequencies for spike and matrix. No envelope targeting was observed. The most diverse response to the peptide pools was observed for subject 38 (Fig. 8C), while subject 48 only had one response (peptide S3, Fig. 8D) that met the criteria for positivity. Surprisingly, four participants who remained negative for SARS-CoV-2 RNA in nasal swab samples between March 23, 2020 and the date of blood collection on April 1, 2021 exhibited cellular responses where at least one peptide probe met the criteria for positivity (Fig. 9). For these samples, nucleocapsid and spike protein region targeting were comparable, and no targeting for matrix and envelope was observed. Subject 24 had the most individual peptide responses meeting the criteria for positivity (Figs. 9C, S1, S7, S8, N2).

## Discussion

Initiated on March 23, 2020, our active workplace clinical surveillance study^18^ has been ongoing for close to two years without interruption. The study has scientific and public health objectives including to: (i) characterize the SARS-CoV-2 dynamics and associated host responses in a small participant cohort monitored intensely longitudinally and over long periods of time; and (ii) provide a safe workplace “bubble”, where the likelihood of SARS-CoV-2 transmission is minimized. Details on the study design have been discussed in detail elsewhere^18^. Testing frequency has varied between three times weekly (Mon, Wed, Fri) and twice weekly (Mon, Thu), depending on Los Angeles County SARS-CoV-2 positivity rates and participant vaccination status. To date, the study successfully has met its primary public health aim, as we have not observed any workplace SARS-CoV-2 transmissions. The current report describes the study’s scientific accomplishments and is focused on nine participants, unvaccinated against SARS-CoV-2 at the time, who developed mild COVID-19 between mid-November, 2020 and mid-March, 2021, before the widespread availability of vaccines.

Nasal swab samples collected from participants who tested positive for SARS-CoV-2 RNA by qPCR were examined by TEM and provided a rare view of the microenvironment contained in nasal swab specimens (Fig. 1). The images show that cells directly bound to the swab tip were frequently ruptured and surrounded by a layer of intact, indirectly bound cells, with little extracellular material between the layers. The majority of cells did not show any signs of infection, and, in the ones that did, unambiguous identification of viral replication organelles and SARS-CoV-2 virions was not possible due to the complexity of cellular structures present in these clinical samples. This illustrates why most TEM images of SARS-CoV-2 originate from cell cultures and occasionally autopsy tissues and, unfortunately, precludes the use of TEM for diagnostic purposes. Our observations are in agreement with Hopfer et al., who also found many confusing cellular structures, in particular, “outside-in” ribosomes within the lumen of the endoplasmic reticulum^22^.

Cultivation of clinical nasal swab specimens containing SARS-CoV-2 RNA using Vero E6 cells, as described previously^25^, did not result in any viral plaque formation according to SARS-CoV-2 nucleocapsid ELISA, even though we routinely use this system to grow laboratory SARS-CoV-2 samples. We speculate that the swab fibers were too efficient at binding and protecting the few viral particles associated with epithelial cells, as supported by our TEM data (Fig. 1A). Attempts to mechanically macerate the swab tip or free the viral cells using sonication were unsuccessful at overcoming these obstacles. We also attempted to generate multiplexed amplicon libraries of clinical specimens for whole-genome sequencing using generation (AmpliSeq, Illumina, San Diego, CA), but the libraries did not meet the necessary quality thresholds. A number of SARS-CoV-2 strains emerged in Southern California in late 2020, spanning the November 2020-March 2021 COVID-19 surge, driven largely by mutations in the spike protein^26^ and described by Zhang et al.^27^. The dominant clades were: 20 A (lineage B.1.234^26^), 20B (lineage B.1.1.220/B.1.1.222^26^), 20 C (lineage B.1.346^28^), and 20 G (lineage B.1.2^26^). Clade CAL.20 C, later assigned the lineage B.1.427/B.1.429^29^, also was gaining prominence over this period^27^.

The high temporal resolution, along with the long-term nature of our small, observational clinical study, has provided previously unreported insights into SARS-CoV-2 in vivo dynamics. The two major phases of the SARS-CoV-2 temporal profiles have been described in terms of viral RNA copy numbers measured by RT-qPCR analysis of nasal swab samples (Figs. 2–4): (i) the rapid, exponential growth phase that often is accompanied by the onset of symptoms (viral proliferation); and (ii) the slow decay phase (viral clearance). For the first time, we report in vivo, clinical SARS-CoV-2 doubling times, *T*_*d*_, (Fig. 3I) in a COVID-19 population, including the transition from test negativity to positivity. The exponential growth phase usually only lasts 3–4 days (Fig. 3), requiring intense longitudinal sampling to capture fully. Unlike with other microorganisms (e.g., generation time in bacterial growth), the complexities of viral replication are not fully captured in a doubling model. However, due to the exponential nature of the early growth kinetics, the *T*_*d*_ value represents a useful mathematical descriptor of viral replication rates, there-by providing a quantity that can be compared across studies. The median *T*_*d*_ value was 3.1 h, and three out of four participants exhibited near identical viral growth kinetics (*T*_*d*_ range, 2.8–3.2 h, Fig. 3I). Interestingly, the one outlier (subject 48, *T*_*d*_ 5.2 h) who experienced slow viral growth kinetics relative to the other three participants also displayed the longest SARS-CoV-2 positivity (Fig. 3B), spanning close to 90 days.

Early SARS-CoV-2 replication kinetics have been measured previously in a laboratory setting and afforded longer *T*_*d*_ values than those measured here using clinical specimens. Cheemarla et al. measured SARS-CoV-2 replication in primary human airway epithelial organoids and reported a *T*_*d*_ value of 5.9 h (95% confidence interval, CI, 4.9–7.4 h)^30^, double the median observed clinically in our study. These authors also calculated a *T*_*d*_ value of 6.5 h based on viral RNA analysis in three clinical samples from one participant (Patient L2)^30^. Killingley et al. characterized the proliferation phase in 18 participants of a human challenge study^16^ but, unfortunately, did not describe the associated kinetics. Ke et al. studied the SARS-CoV-2 dynamics during acute infection through a daily longitudinal sampling of 60 individuals at the University of Illinois at Urbana-Champaign^31^. As in the current report, the investigators captured both the rise and decline of viral shedding in nasal and/or saliva samples and found the patterns to be highly heterogeneous across participants. These results are highly complementary to those reported here.

We conducted a model analysis of the *C*_*t*_ values from the above nasal swab samples using a range of approaches (Fig. 4, Supplementary Figs. 1 and 2). Summary data presented in Table 2 show that derived kinetic parameters based on different priors (informative, uninformative, and biased) are largely consistent. Our modeled proliferation times (3.1 d, informative priors; the model approach most consistent with our experimental observations for this phase) closely agreed with Kissler et al.^8^ (3.2 d overall), but considerably shorter than those observed in a human challenge study (6.2 d)^16^. We estimated a longer mean clearance time (15.3–17.2 d, depending on the model, versus 8.5 d overall for Kissler et al.^8^) and a much lower mean peak *C*_*t*_ value (i.e., higher viral titer) from our dataset, compared to the results reported by Kissler et al*.*^8^ (Table 2). The participants in the study by Kissler et al. consisted National Basketball Association (NBA) members, including elite athletes with physiology that differs from our study participants. There also could be bias introduced based on the sampling frequency. Our study is designed to identify all cases of infection by intense longitudinal sampling for all employees. In this way, we can detect early infection events that could have been missed in Kissler et al., possibly explaining our longer clearance time and lower mean peak *C*_*t*_ values.

Our study also characterized the host immune dynamics (using cytokine, antibody, and CD8^+^ T-cell activity) in response to mild COVID-19 infection. As with other studies^7,32^, cytokine concentration-time profiles (Fig. 5) were heterogeneous across individuals. The nature of the inflammatory markers providing quantifiable temporal profiles differed across studies. Collectively, these data suggest that the predictive and mechanistic role of these signaling proteins in COVID-19 patients remains poorly understood. Temporal profiles of serum antibody responses to SARS-CoV-2 also were highly heterogenous across study participants. We successfully measured the decay half-lives of the shorter-lived antibodies, IgA and IgM (Fig. 7), and found them to be statistically equivalent, while IgG concentrations remained stable for most participants over the *ca*. 130 days of observation (Fig. 6). Although several participants exhibited some decay of the IgG response, our results contrasted with prior findings using the same methodology that showed more rapid decay up to the first 90–120 days^19,33^. However, recent large studies have analyzed the long-term (>1 year), longitudinal, humoral neutralizing activity in response to natural SARS-CoV-2 infection and found the titers to be stable over time, especially in non-hospitalized individuals^34,35^. These results agree with our observations.

Cellular CD8^+^ T-cell response profiles targeting SARS-CoV-2 differed across participants who became positive for SARS-CoV-2 RNA by RT-qPCR 2–4 months earlier, but generally had the highest frequency response to the nucleocapsid peptide pool, compared to spike, matrix, and envelope protein regions. This was similar to findings in a cohort of persons tested *ca*. 30 days after the onset of COVID-19 symptoms^20^.

There are some limitations associated with the results from our study that followed a small cohort of nine participants who developed mild COVID-19 symptoms. Viral RNA quantified in clinical specimens by RT-qPCR was used as a proxy for SARS-CoV-2 shedding, but viral genome copies would not necessarily reflect titers of infectious viruses. Evaluating viral load by plaque assay would have been challenging given the difficulties in isolating the virus from clinical swab samples, as described above. Model estimates of viral proliferation time (Table 2) need to be interpreted with caution as they are sparely sampled when compared to the clearance phase^8^. Caveats pertaining to the analysis of serum antibody^18,19^ and cellular responses^20^ have been discussed elsewhere, but include the considerations that RBD-binding antibodies may not correspond to neutralizing activity, and the limitations of using synthetic overlapping peptides to map T-cell responses.

Our clinical study produced a number of unexpected results. One participant (subject 48), believed to have been exposed to SARS-CoV-2 on December 25, 2020, started to experience symptoms consistent with COVID-19 on December 27 (Sunday) and tested positive for viral RNA by RT-qPCR in a nasal swab sample the following day (Monday) at a scheduled test. The low *C*_*t*_ values measured on December 28 (N1, 15.36; N2, 16.31) corresponding to a viral load of 9.0 × 10^7^ copies per swab (Fig. 3B) suggest this individual likely would have been infectious the previous day when symptoms first presented, only two days following exposure. Another participant (subject 38) tested negative for SARS-CoV-2 RNA on December 28, 2020, but experienced symptoms consistent with COVID-19 the following day and tested positive a day later, on December 30, with low *C*_*t*_ values (N1, 16.56; N2, 18.17) and viral loads of 3.2 × 10^7^ copies per swab (Fig. 3A). As with subject 48, the viral proliferation trajectory suggests that this participant would have been infectious the previous day (December 29) coinciding with the onset of symptoms, and only 1 day after a negative test result. These results contradict the dogma that COVID-19 symptoms manifest following an infectious, asymptomatic phase spanning multiple days. Furthermore, viral replication following exposure is so rapid (*T*_*d*_ *ca*. 3 h) that a negative test result may only provide a one-day safe window prior to becoming infectious.

One participant (subject 82) developed asymptomatic COVID-19 as evidenced by low, sporadic viral RNA copies in nasal samples for a short duration (Fig. 7), similar to subject 31 in our previous report^18^. Subject 82 was exposed to two household members with mild COVID-19, and we were surprised initially that the participant did not develop similar viral load kinetics, punctuated by high *C*_*max*_ values. However, serum IgG concentrations were constant between *ca*. 100–200 ng mL^−1^ (Fig. 7), even preceding the first positive test result, indicating that the unvaccinated participant had acquired some level of immunity from previous, unknown exposure.

Finally, CD8^+^ T-cell targeting of SARS-CoV-2 in blood samples from participants who did not become positive for SARS-CoV-2 RNA by RT-qPCR between March 23, 2020 (study start date) and April 1, 2021 (date of blood collection) revealed that at least four individuals met the criteria for positivity (i.e., likely prior exposure to SARS-CoV-2 eliciting an immune response, Fig. 9). Subjects 17 and 18 had serum IgG (1.85 µg mL^−1^) and IgM^18^ concentrations, respectively, at study baseline (subject 17, April 3, 2020; subject 18, March 27, 2020) suggestive of COVID-19 predating the start of the clinical surveillance study. Subject 54 (Fig. 9D) was exposed to household members with COVID-19 on several occasions but did not test positive for SARS-CoV-2 RNA by RT-qPCR, likely due to pre-existing immunity.

There are few reports capturing the kinetics describing the complete SARS-CoV-2 growth cycle (*i.e*., viral proliferation and clearance) in a clinical setting, along with the concomitant host responses. Vetter et al. monitored viral shedding and the adaptive immune responses of five COVID-19 patients in Geneva, Switzerland^7^. Two similar reports studied the SARS-CoV-2 viral load dynamics in specimens collected from a range of anatomic sites along with serum antibody responses for 23 hospitalized patients in Hong Kong^6,36^. In prospective clinical studies, Grad and co-workers measured the longitudinal viral RNA trajectories in NBA members^8,24,37^. The investigators modeled the time to *C*_*max*_ and the viral clearance phase. A modeling study analyzing the nasopharyngeal SARS-CoV-2 viral kinetics of 655 hospitalized patients in France provided valuable associations between patient characteristics and mortality, but identified a lack of data for the first days from symptom onset as a limitation^9^. Our study is highly complementary to the above reports and helps address some of the gaps relating to viral kinetics in the proliferation phase and diversity of patient populations, both demographically and in terms of COVID-19 symptom severity.

In conclusion, the current report demonstrates that our small, workplace, longitudinal clinical surveillance study exceeded its original objectives: preventing workplace SARS-CoV-2 transmissions, over nearly two years and spanning multiple COVID-19 surges, and elucidating the clinical biology of the virus. It also shows that intense, long-term, repeat-sampling of the same group of unvaccinated participants (during the study phase reported here) has led to a number of scientific accomplishments rooted in a description of the kinetics encompassing the phases of the SARS-CoV-2 clinical life-cycle, and the dynamics of the associated host immune responses. Our results underscore the highly heterogeneous nature of these processes across individuals. We hope that these results can play a role in supporting the development of guidelines for the clinical management of COVID-19 patients^38^ as well as improving public health policies (e.g., the onset of symptoms relative to viral replication cycle, viral growth and clearance kinetics and isolation guidelines, recommended qPCR testing frequency).

## Supplementary information

Description of Additional Supplementary Files

Supplementary Information

Supplementary Data 1

Supplementary Data 2

Supplementary Data 3

Supplementary Data 4

Reporting Summary

## Data Availability

The source data for Figs. 2, 3 and 7 can be found in Supplementary Data 1. The source data for Fig. 5 can be found in Supplementary Data 2. The source data for Figs. 6 and 7 can be found in Supplementary Data 3. The source data for Figs. 8 and 9 can be found in Supplementary Data 4.
