# Supplementary material for: Early SARS-CoV-2 dynamics and immune responses in unvaccinated participants of an intensely sampled longitudinal surveillance study
Source: Commun Med (Lond). 2022 Oct 11;2:129. doi: 10.1038/s43856-022-00195-4 (PMC9553075; doi:10.1038/s43856-022-00195-4)
Supplement: Supplementary file 1 — Description of Additional Supplementary Files [file 43856_2022_195_MOESM1_ESM.pdf]

Description of Additional Supplementary Files

File Name: Supplementary Data 1

Description: Longitudinal nasal swab SARS-CoV-2 viral load (qPCR) kinetics dataset (XLS)

File Name: Supplementary Data 2

Description: Cytokine/chemokine concentration-time profile dataset for participants testing positive nasally for SARS-CoV-2 RNA (XLS)

File Name: Supplementary Data 3

Description: Longitudinal antibody responses to SARS-CoV-2 in serum samples. Dataset for six participants testing positive for SARS-CoV-2 by qPCR (XLS)

File Name: Supplementary Data 4

Description: Evaluation of $CD8^+$ T cell targeting of SARS-CoV-2 in blood samples. Dataset for all participants tested (XLS)
