# Supplementary material for: Early SARS-CoV-2 dynamics and immune responses in unvaccinated participants of an intensely sampled longitudinal surveillance study
Source: Commun Med (Lond). 2022 Oct 11;2:129. doi: 10.1038/s43856-022-00195-4 (PMC9553075; doi:10.1038/s43856-022-00195-4)
Supplement: Supplementary file 2 — Supplementary Information [file 43856_2022_195_MOESM2_ESM.pdf]

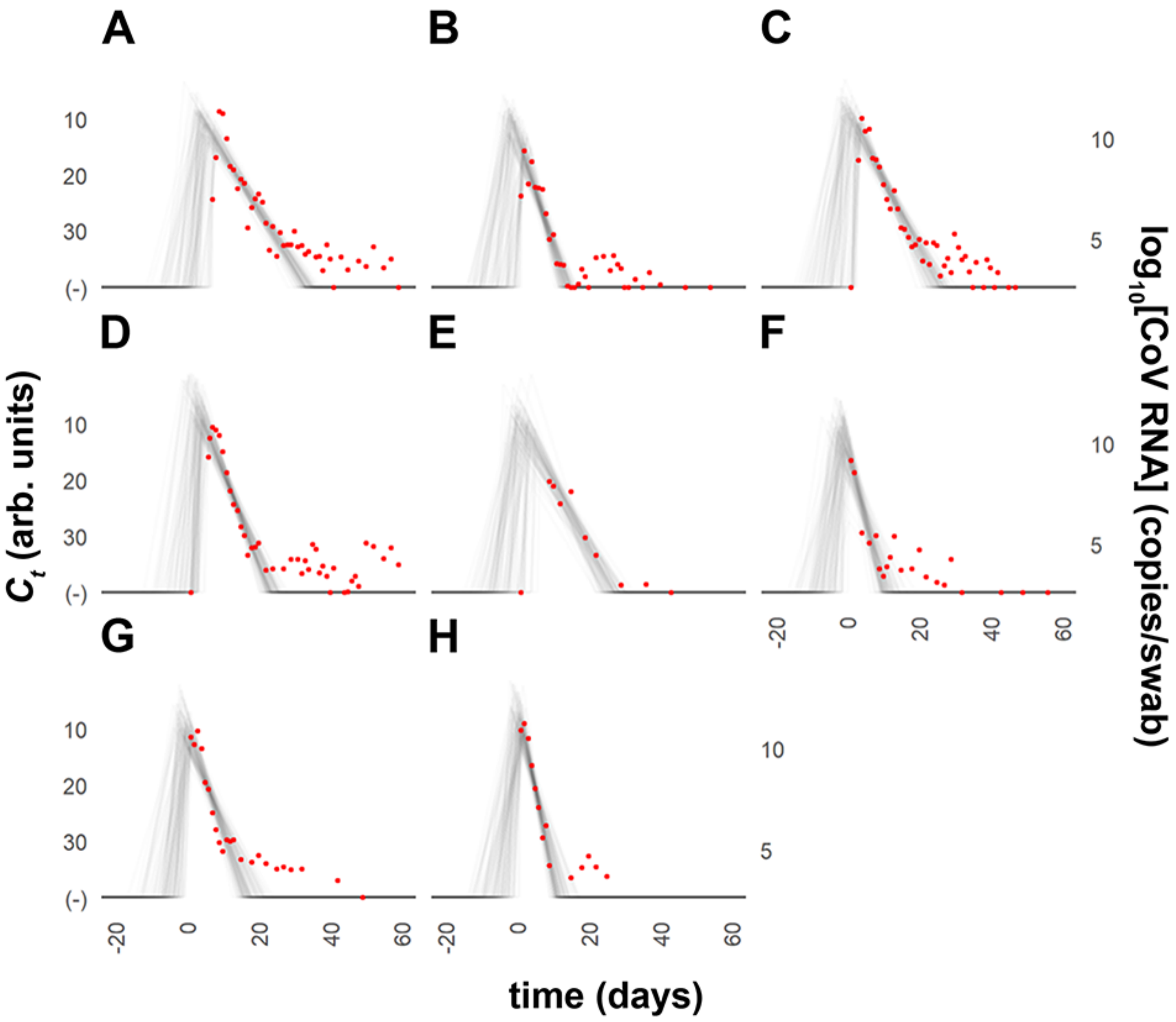

Supplementary Fig. 1. Uninformative priors model plots (grey) for C_t values and estimated trajectories for infections; each red circle corresponds to one observation; T (d) indicates the time since the minimum C_t value (highest viral load).

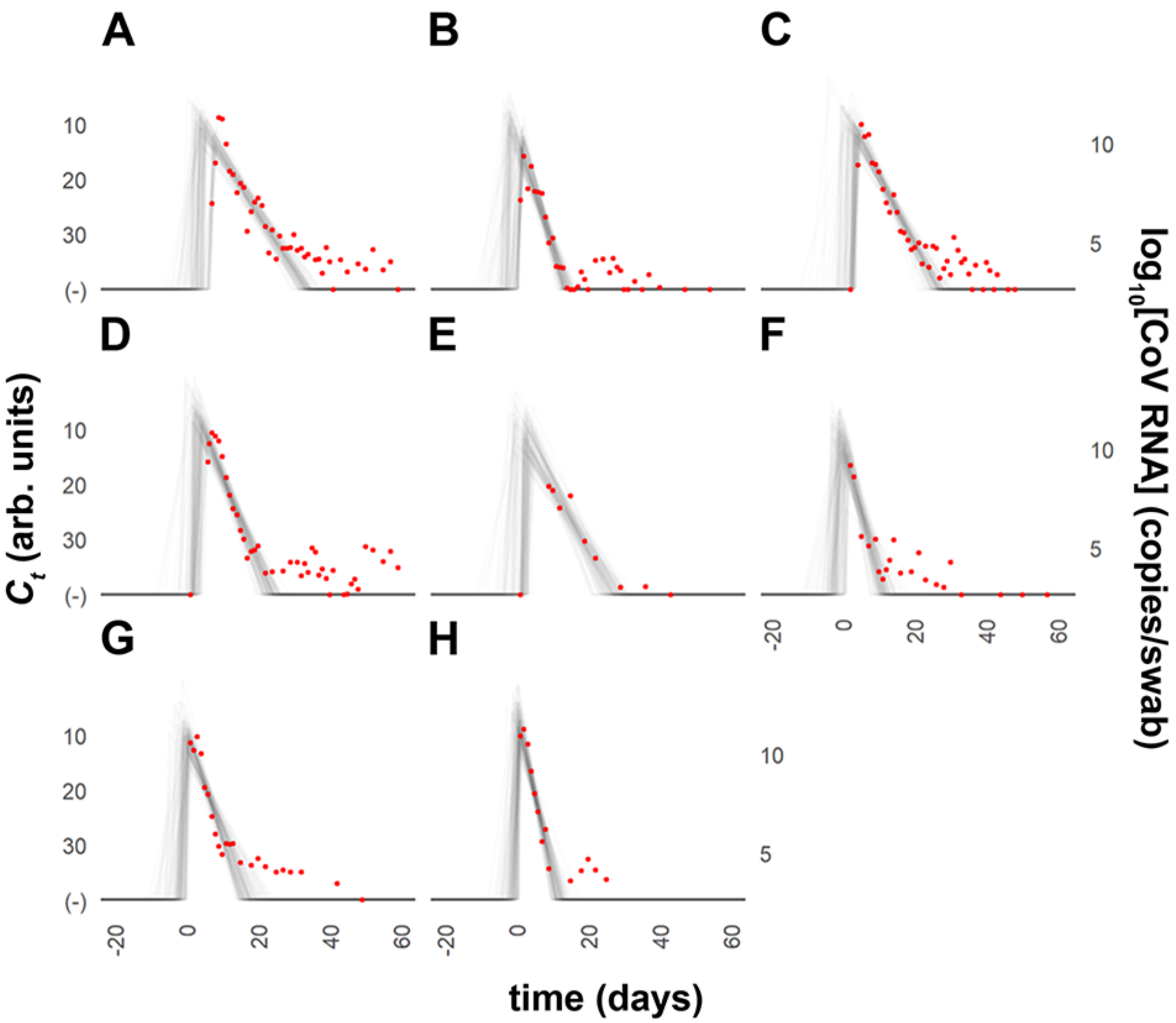

Supplementary Fig. 2. Biased set of priors model plots (grey) for C_t values and estimated trajectories for infections; each red circle corresponds to one observation; T (d) indicates the time since the minimum C_t value (highest viral load).

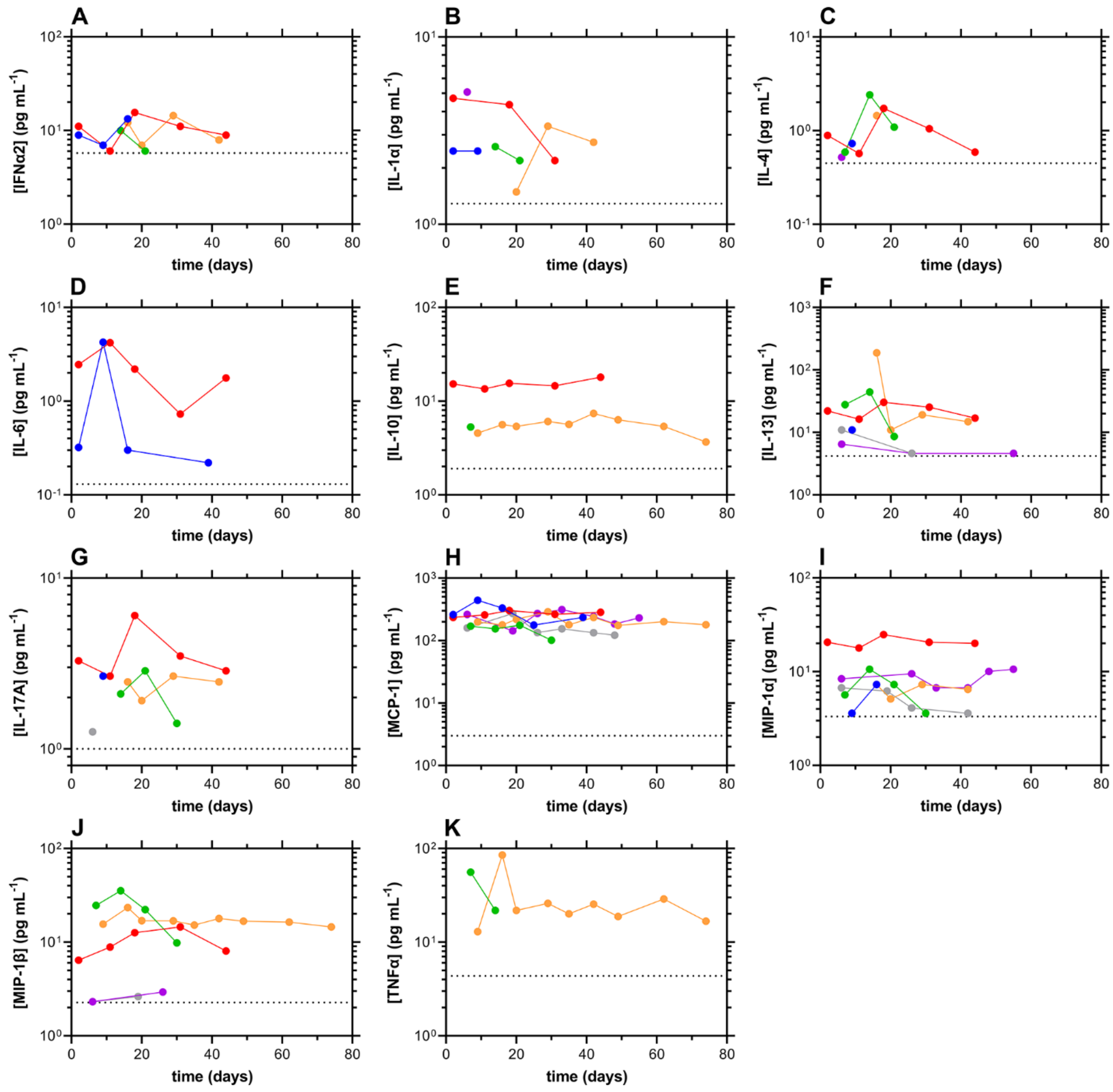

Supplementary Fig. 3. Cytokine/chemokine concentration-time profiles for participants testing positive nasally for SARS-CoV-2 RNA, normalized temporally to the first day of positivity. Not all subjects testing positive participated in the blood collection portion of the study . The dotted line represents the assay lower limit of quantitation; blue, subject 2; red, subject 21; green, subject 38; orange, subject 48; magenta, subject 83; grey, subject 84. (**A**) IFNα2; (**B**) IL-1α; (**C**) IL-4; (**D**) IL-6; (**E**) IL-10; (**F**) IL-13; (**G**) IL-17A; (**H**) MCP-1; (**I**) MIP-1α; (**J**) MIP-1β; (**K**) TNFα.
